# (*E*)-*N*′-(2-Hy­droxy­benzyl­idene)furan-2-carbohydrazide

**DOI:** 10.1107/S1600536810024839

**Published:** 2010-07-14

**Authors:** Rahman Bikas, Hassan Hosseini Monfared, Canan Kazak, N. Burcu Arslan, Keyvan Bijanzad

**Affiliations:** a Department of Chemistry, Zanjan University, 45195-313 Zanjan, Iran; b Department of Physics, Faculty of Arts and Sciences, Ondokuz Mayis University, 55019 Kurupelit, Samsun, Turkey; c Faculty of Chemistry, Iran University of Science and Technology (IUST), 16846 Tehran, Iran

## Abstract

In the title compound, C_12_H_10_N_2_O_3_, the dihedral angle between the benzene ring and the furan ring is 16.12 (13)°. The conformation is stabilized by an intra­molecular O—H⋯N hydrogen bond. Inter­molecular N—H⋯O hydrogen bonds with the keto group as acceptor lead to strands along [001]. The mol­ecule displays a *trans* configuration with respect to the C=N and N—N bonds.

## Related literature

For historical background to aroylhydrazones, see: Offe *et al.* (1952 ▶); Craliz *et al.* (1955 ▶); Pickart *et al.* (1983 ▶); Arapov *et al.* (1987 ▶); Ranford *et al.* (1998 ▶); Savanini *et al.* (2002 ▶). For related structures, see: Monfared *et al.* (2010 ▶); Ali *et al.* (2005 ▶); Li *et al.* (2007 ▶); Diao *et al.* (2007 ▶).
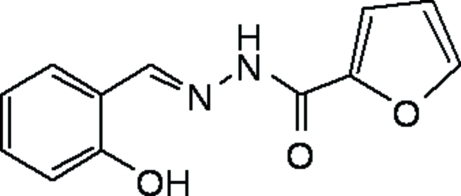


## Experimental

### 

#### Crystal data

C_12_H_10_N_2_O_3_
*M* *_r_* = 230.22
Orthorhombic, 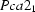

*a* = 17.3539 (15) Å
*b* = 6.3320 (4) Å
*c* = 9.8613 (7) Å
*V* = 1083.61 (14) Å^3^
*Z* = 4
Mo *K*α radiation
μ = 0.10 mm^−1^
*T* = 293 K
0.46 × 0.29 × 0.20 mm

#### Data collection

Stoe IPDS 2 diffractometer
6705 measured reflections
1130 independent reflections
792 reflections with *I* > 2σ(*I*)
*R*_int_ = 0.056

#### Refinement

*R*[*F* ^2^ > 2σ(*F* ^2^)] = 0.027
*wR*(*F* ^2^) = 0.043
*S* = 0.81
1130 reflections
159 parameters
3 restraints
H atoms treated by a mixture of independent and constrained refinement
Δρ_max_ = 0.10 e Å^−3^
Δρ_min_ = −0.11 e Å^−3^

### 

Data collection: *X-AREA* (Stoe & Cie, 2002 ▶); cell refinement: *X-AREA*; data reduction: *X-RED32* (Stoe & Cie, 2002 ▶); program(s) used to solve structure: *SHELXS97* (Sheldrick, 2008 ▶); program(s) used to refine structure: *SHELXL97* (Sheldrick, 2008 ▶); molecular graphics: *ORTEP-3 for Windows* (Farrugia, 1997 ▶); software used to prepare material for publication: *WinGX* (Farrugia, 1999 ▶).

## Supplementary Material

Crystal structure: contains datablocks I, global. DOI: 10.1107/S1600536810024839/vm2028sup1.cif

Structure factors: contains datablocks I. DOI: 10.1107/S1600536810024839/vm2028Isup2.hkl

Additional supplementary materials:  crystallographic information; 3D view; checkCIF report

## Figures and Tables

**Table 1:** Hydrogen-bond geometry (Å, °)

*D*—H⋯*A*	*D*—H	H⋯*A*	*D*⋯*A*	*D*—H⋯*A*
N2—H2⋯O2^i^	0.86	2.21	2.900 (2)	137
O1—H22⋯N1	0.90 (3)	1.89 (3)	2.651 (3)	141 (3)

Symmetry code: (i) 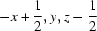.

**Fig. 1.**
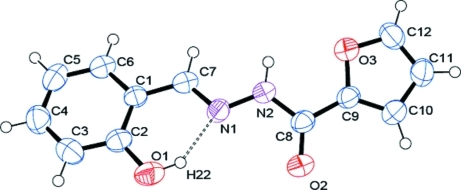
The molecular structure of the title compound, with atom labels and anisotropic displacement ellipsoids (drawn at 50% probability level) for non-H atoms. The dashed line indicates intramolecular O–H···N hydrogen bond.

**Fig. 2.**
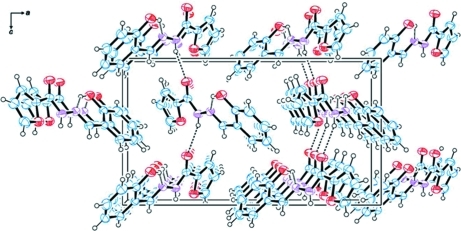
View of the unit cell of the title compound viewed along [010]. Hydrogen bonds are drawn as dashed lines.

## supplementary crystallographic information

### Comment

As part of our studies on the synthesis and characterization of aroylhydrazone derivatives, we report the crystal structure of (*E*)—*N*'-(2-hydroxybenzylidene)furan-2-carbohydrazide.

The asymmetric unit contains one molecule of the title compound, which is shown in Figure 1. The molecule is almost planar with a dihedral angle of 16.12 (13)° between the benzene ring and the furan ring. This configuration is stabilized by an intramolecular O—H···N hydrogen bond, with the nitrogen of the azomethine group (–C=N–) acting as acceptor. Intermolecular N—H···O hydrogen bonds with the keto group as acceptor lead to strands along [001] (Fig. 2).

### Experimental

All reagents were commercially available and used as received. A methanol (10 ml) solution of 2-hydroxybenzaldehyde (1.5 mmol) was drop-wise added to a methanol solution (10 ml) of 2-furanecarboxylic acid hydrazide (1.5 mmol), and the mixture was refluxed for 3 h. Then the solution was evaporated on a steam bath to 5 cm^3^ and cooled to room temperature. Light yellow precipitates of the title compound were separated and filtered off, washed with 3 ml of cooled methanol and then dried in air. X-ray quality crystals of the title compound were obtained from methanol by slow solvent evaporation. Yield: 78%, mp 191 °C.

### Refinement

O– and N–bound H atoms were refined freely. C-bonded H atoms were positioned geometrically (C—H = 0.93 Å) and treated as riding on their parent atoms [*U*_iso_(H) = 1.2*U*_eq_(C)]. 9672 Friedel pairs have been merged. The Flack parameter is meaningless.

### Crystal data

**Table:**

C_12_H_10_N_2_O_3_	*F*(000) = 480
*M**_r_* = 230.22	*D*_x_ = 1.411 Mg m^−^^3^
Orthorhombic, *P**c**a*2_1_	Mo *K*α radiation, λ = 0.71073 Å
Hall symbol: P 2c -2ac	Cell parameters from 6480 reflections
*a* = 17.3539 (15) Å	θ = 2.1–26.9°
*b* = 6.3320 (4) Å	µ = 0.10 mm^−^^1^
*c* = 9.8613 (7) Å	*T* = 293 K
*V* = 1083.61 (14) Å^3^	Prism, light yellow
*Z* = 4	0.46 × 0.29 × 0.20 mm

### Data collection

**Table:**

Stoe IPDS 2 diffractometer	792 reflections with *I* > 2σ(*I*)
Radiation source: fine-focus sealed tube	*R*_int_ = 0.056
graphite	θ_max_ = 26.0°, θ_min_ = 2.4°
Detector resolution: 6.67 pixels mm^-1^	*h* = −21→21
w scans	*k* = −6→7
6705 measured reflections	*l* = −11→12
1130 independent reflections	

### Refinement

**Table:**

Refinement on *F*^2^	Primary atom site location: structure-invariant direct methods
Least-squares matrix: full	Secondary atom site location: difference Fourier map
*R*[*F*^2^ > 2σ(*F*^2^)] = 0.027	Hydrogen site location: inferred from neighbouring sites
*wR*(*F*^2^) = 0.043	H atoms treated by a mixture of independent and constrained refinement
*S* = 0.81	*w* = 1/[σ^2^(*F*_o_^2^) + (0.0138*P*)^2^] where *P* = (*F*_o_^2^ + 2*F*_c_^2^)/3
1130 reflections	(Δ/σ)_max_ < 0.001
159 parameters	Δρ_max_ = 0.10 e Å^−^^3^
3 restraints	Δρ_min_ = −0.11 e Å^−^^3^

### Special details

**Table:**

Geometry. All e.s.d.'s (except the e.s.d. in the dihedral angle between two l.s. planes) are estimated using the full covariance matrix. The cell e.s.d.'s are taken into account individually in the estimation of e.s.d.'s in distances, angles and torsion angles; correlations between e.s.d.'s in cell parameters are only used when they are defined by crystal symmetry. An approximate (isotropic) treatment of cell e.s.d.'s is used for estimating e.s.d.'s involving l.s. planes.
Refinement. Refinement of *F*^2^ against ALL reflections. The weighted *R*-factor *wR* and goodness of fit *S* are based on *F*^2^, conventional *R*-factors *R* are based on *F*, with *F* set to zero for negative *F*^2^. The threshold expression of *F*^2^ > σ(*F*^2^) is used only for calculating *R*-factors(gt) *etc*. and is not relevant to the choice of reflections for refinement. *R*-factors based on *F*^2^ are statistically about twice as large as those based on *F*, and *R*- factors based on ALL data will be even larger.

### Fractional atomic coordinates and isotropic or equivalent isotropic displacement parameters (Å^2^)

**Table:**

	*x*	*y*	*z*	*U*_iso_*/*U*_eq_	
C1	0.44074 (10)	0.4371 (3)	0.5468 (2)	0.0396 (5)	
C2	0.43426 (12)	0.2756 (4)	0.6432 (2)	0.0459 (6)	
C3	0.48301 (13)	0.1039 (4)	0.6372 (3)	0.0594 (7)	
H3	0.4781	−0.0039	0.7006	0.071*	
C4	0.53899 (13)	0.0898 (4)	0.5384 (3)	0.0616 (7)	
H4	0.5710	−0.0278	0.5349	0.074*	
C5	0.54754 (12)	0.2496 (4)	0.4452 (3)	0.0579 (7)	
H5	0.5859	0.2417	0.3796	0.070*	
C6	0.49918 (11)	0.4207 (4)	0.4496 (3)	0.0496 (6)	
H6	0.5054	0.5285	0.3865	0.060*	
C7	0.38950 (11)	0.6177 (4)	0.5438 (2)	0.0461 (6)	
H7	0.3928	0.7123	0.4718	0.055*	
C8	0.25245 (13)	0.9022 (4)	0.7261 (2)	0.0447 (5)	
C9	0.21197 (12)	1.0979 (4)	0.6958 (2)	0.0449 (6)	
C10	0.16454 (13)	1.2205 (4)	0.7684 (3)	0.0569 (6)	
H10	0.1472	1.1948	0.8561	0.068*	
C11	0.14591 (15)	1.3964 (4)	0.6865 (3)	0.0626 (8)	
H11	0.1139	1.5085	0.7097	0.075*	
C12	0.18302 (13)	1.3702 (4)	0.5694 (3)	0.0607 (7)	
H12	0.1808	1.4634	0.4966	0.073*	
N1	0.33997 (9)	0.6505 (3)	0.63712 (19)	0.0455 (5)	
N2	0.29468 (9)	0.8264 (3)	0.62150 (19)	0.0489 (5)	
H2	0.2931	0.8892	0.5442	0.059*	
O1	0.38068 (10)	0.2824 (3)	0.74331 (17)	0.0594 (5)	
O2	0.24940 (11)	0.8172 (2)	0.83793 (16)	0.0575 (4)	
O3	0.22438 (8)	1.1887 (3)	0.57132 (16)	0.0557 (4)	
H22	0.3504 (17)	0.397 (4)	0.736 (3)	0.095 (11)*	

### Atomic displacement parameters (Å^2^)

**Table:**

	*U*^11^	*U*^22^	*U*^33^	*U*^12^	*U*^13^	*U*^23^
C1	0.0403 (11)	0.0426 (14)	0.0360 (12)	−0.0038 (10)	−0.0032 (11)	−0.0029 (12)
C2	0.0445 (12)	0.0538 (16)	0.0395 (13)	−0.0066 (11)	−0.0031 (12)	−0.0030 (13)
C3	0.0668 (15)	0.0509 (16)	0.0604 (17)	0.0002 (14)	−0.0073 (15)	0.0084 (14)
C4	0.0566 (14)	0.0627 (18)	0.0653 (18)	0.0127 (13)	−0.0034 (15)	−0.0040 (18)
C5	0.0463 (14)	0.074 (2)	0.0536 (16)	0.0036 (13)	0.0022 (13)	−0.0168 (15)
C6	0.0493 (14)	0.0584 (16)	0.0412 (15)	−0.0033 (13)	0.0019 (11)	0.0001 (15)
C7	0.0469 (11)	0.0547 (16)	0.0366 (12)	−0.0040 (11)	0.0014 (12)	0.0014 (11)
C8	0.0444 (12)	0.0533 (14)	0.0364 (13)	0.0000 (12)	−0.0023 (10)	−0.0041 (14)
C9	0.0428 (12)	0.0559 (15)	0.0360 (13)	0.0037 (12)	−0.0005 (10)	−0.0039 (13)
C10	0.0599 (14)	0.0665 (18)	0.0444 (14)	0.0099 (15)	0.0048 (13)	−0.0046 (14)
C11	0.0652 (17)	0.0651 (18)	0.0576 (17)	0.0197 (14)	−0.0024 (13)	−0.0115 (16)
C12	0.0649 (15)	0.0568 (19)	0.0604 (17)	0.0135 (13)	−0.0125 (14)	0.0000 (15)
N1	0.0450 (10)	0.0506 (13)	0.0409 (10)	0.0042 (9)	0.0020 (10)	−0.0045 (10)
N2	0.0587 (10)	0.0532 (12)	0.0349 (11)	0.0110 (10)	0.0020 (10)	−0.0009 (10)
O1	0.0615 (10)	0.0708 (13)	0.0459 (10)	−0.0058 (10)	0.0095 (9)	0.0086 (10)
O2	0.0702 (10)	0.0643 (10)	0.0382 (10)	0.0069 (9)	0.0059 (9)	0.0018 (9)
O3	0.0592 (10)	0.0644 (11)	0.0434 (10)	0.0118 (8)	0.0015 (8)	0.0016 (10)

### Geometric parameters (Å, °)

**Table:**

C1—C6	1.399 (3)	C8—O2	1.228 (3)
C1—C2	1.401 (3)	C8—N2	1.354 (3)
C1—C7	1.449 (3)	C8—C9	1.456 (3)
C2—O1	1.357 (3)	C9—C10	1.339 (3)
C2—C3	1.379 (3)	C9—O3	1.372 (3)
C3—C4	1.378 (3)	C10—C11	1.413 (3)
C3—H3	0.9300	C10—H10	0.9300
C4—C5	1.375 (4)	C11—C12	1.332 (4)
C4—H4	0.9300	C11—H11	0.9300
C5—C6	1.371 (3)	C12—O3	1.355 (3)
C5—H5	0.9300	C12—H12	0.9300
C6—H6	0.9300	N1—N2	1.371 (2)
C7—N1	1.277 (3)	N2—H2	0.8600
C7—H7	0.9300	O1—H22	0.90 (3)
			
C6—C1—C2	117.9 (2)	O2—C8—N2	123.5 (2)
C6—C1—C7	119.3 (2)	O2—C8—C9	122.5 (2)
C2—C1—C7	122.75 (19)	N2—C8—C9	114.0 (2)
O1—C2—C3	118.5 (2)	C10—C9—O3	109.4 (2)
O1—C2—C1	121.7 (2)	C10—C9—C8	132.8 (2)
C3—C2—C1	119.8 (2)	O3—C9—C8	117.72 (19)
C4—C3—C2	120.9 (2)	C9—C10—C11	106.9 (2)
C4—C3—H3	119.5	C9—C10—H10	126.5
C2—C3—H3	119.5	C11—C10—H10	126.5
C5—C4—C3	120.0 (2)	C12—C11—C10	106.7 (2)
C5—C4—H4	120.0	C12—C11—H11	126.7
C3—C4—H4	120.0	C10—C11—H11	126.7
C6—C5—C4	119.6 (2)	C11—C12—O3	110.5 (2)
C6—C5—H5	120.2	C11—C12—H12	124.8
C4—C5—H5	120.2	O3—C12—H12	124.8
C5—C6—C1	121.6 (2)	C7—N1—N2	115.92 (19)
C5—C6—H6	119.2	C8—N2—N1	120.84 (19)
C1—C6—H6	119.2	C8—N2—H2	119.6
N1—C7—C1	121.8 (2)	N1—N2—H2	119.6
N1—C7—H7	119.1	C2—O1—H22	111.8 (19)
C1—C7—H7	119.1	C12—O3—C9	106.5 (2)
			
C6—C1—C2—O1	178.0 (2)	N2—C8—C9—C10	179.1 (2)
C7—C1—C2—O1	−2.3 (3)	O2—C8—C9—O3	174.7 (2)
C6—C1—C2—C3	−2.2 (3)	N2—C8—C9—O3	−3.9 (3)
C7—C1—C2—C3	177.5 (2)	O3—C9—C10—C11	0.1 (3)
O1—C2—C3—C4	−179.3 (2)	C8—C9—C10—C11	177.4 (2)
C1—C2—C3—C4	0.9 (3)	C9—C10—C11—C12	0.0 (3)
C2—C3—C4—C5	0.9 (4)	C10—C11—C12—O3	−0.1 (3)
C3—C4—C5—C6	−1.2 (4)	C1—C7—N1—N2	−179.55 (19)
C4—C5—C6—C1	−0.2 (3)	O2—C8—N2—N1	−2.7 (3)
C2—C1—C6—C5	1.9 (3)	C9—C8—N2—N1	175.82 (18)
C7—C1—C6—C5	−177.8 (2)	C7—N1—N2—C8	−165.59 (19)
C6—C1—C7—N1	−173.37 (19)	C11—C12—O3—C9	0.2 (3)
C2—C1—C7—N1	6.9 (3)	C10—C9—O3—C12	−0.2 (2)
O2—C8—C9—C10	−2.3 (4)	C8—C9—O3—C12	−177.90 (18)

### Hydrogen-bond geometry (Å, °)

**Table:**

*D*—H···*A*	*D*—H	H···*A*	*D*···*A*	*D*—H···*A*
N2—H2···O2^i^	0.86	2.21	2.900 (2)	137
O1—H22···N1	0.90 (3)	1.89 (3)	2.651 (3)	141 (3)

Symmetry codes: (i) −*x*+1/2, *y*, *z*−1/2.
